# Calcium Crystal Deposits in Kidney Allografts: The Interplay of PTH Levels and Phosphaturia

**DOI:** 10.1016/j.xkme.2026.101464

**Published:** 2026-07-11

**Authors:** Simona Barbuto, Gisella Vischini, Lorenza Magagnoli, Michele Provenzano, Gian Marco Berti, Angelodaniele Napoletano, Mario Gennaro Cozzolino, Giorgia Comai, Gaetano La Manna, Giuseppe Cianciolo

**Affiliations:** 1 Nephrology, Dialysis and Kidney Transplant Unit, IRCCS Azienda Ospedaliero-Universitaria di Bologna, Bologna, Italy; 2 Department of Health Sciences, University of Milan, Milan, Italy; 3 Renal Division, ASST Santi Paolo e Carlo, Milan, Italy; 4 Department of Pharmacy, Health and Nutritional Sciences, University of Calabria, Rende, Cosenza, Italy; 5 Department of Medical and Surgical Science (DIMEC), Alma Mater Studiorum - University of Bologna, Bologna, Italy

**Keywords:** kidney transplantation, hyperparathyroidism, parathyroid hormone (PTH), nephrocalcinosis, calcium crystal deposits, phosphate wasting

## Abstract

**Rationale & Objective:**

Hyperparathyroidism is common in kidney transplant recipients and may contribute to graft injury through calcium crystal deposition. We investigated the association between pretransplant parathyroid hormone (PTH) levels and calcium crystal deposits (CCDs) and whether fractional excretion of phosphate (FePi) mediates this relationship.

**Study Design:**

Retrospective single-center observational study.

**Setting & Participants:**

Forty-one kidney transplant recipients undergoing protocol biopsies at 3 and 12 months posttransplantation.

**Exposure:**

Pretransplant PTH levels.

**Outcome(s):**

Presence of calcium crystal deposits in graft biopsies.

**Analytical Approach:**

Logistic regression models and mediation analysis were used to evaluate associations between PTH levels, FePi, and CCDs.

**Results:**

CCDs were detected in 33% of biopsies at 3 months and 45% at 12 months. Higher pretransplant PTH levels were associated with increased odds of CCDs. Mediation analysis showed that approximately 72% of this effect was explained using FePi levels. At 24 months posttransplantation, no meaningful difference in estimated glomerular filtration rate was observed between patients with and without CCDs.

**Limitations:**

Retrospective design, small sample size, absence of fibroblast growth factor 23 level measurements, and limited adjustment for potential confounders.

**Conclusions:**

Elevated pretransplant PTH levels are associated with calcium crystal deposition in kidney allografts, largely mediated by phosphate wasting. However, no clear impact on graft function was observed within the first 2 years posttransplantation.

## Plain-Language Summary

After kidney transplantation, disturbances in mineral metabolism are common and may contribute to subtle damage to the transplanted kidneys. In this study, we explored whether high levels of parathyroid hormone before transplantation are linked to the formation of calcium crystal deposits in the kidney graft. We analyzed biopsy samples collected after transplantation and examined how the kidney handles phosphate. We found that higher parathyroid hormone levels were associated with the presence of these deposits, largely through increased loss of phosphate in the urine. However, these deposits did not appear to affect kidney function within the first 2 years after transplantation. These findings suggest that better control of mineral metabolism before transplantation may help reduce early changes in the kidney graft.

## Introduction

Transplantation is the most effective treatment for patients with kidney failure because it reduces cardiovascular risk, improves quality of life, and lowers health care economic costs. Unfortunately, alterations related to chronic kidney disease (CKD)-mineral and bone disorder persist, and among these, posttransplant hyperparathyroidism (HPT) plays a pivotal role.^1^ Despite the improvement in parathyroid hormone (PTH) level after recovery of kidney function and the rapid reduction in the 3 months post-kidney transplant (post-KT), a small percentage of kidney transplant recipients (KTRs) reach values within the normal range.^2^

Posttransplant HPT can be differentiated into a maladaptive response (“persistent HPT,” PHPT) versus a compensatory adaptive response (“de novo” HPT). PHPT occurs as a result of pre-existing secondary HPT, which can further worsen posttransplant outcomes.^3^^,^^4^ Conversely, de novo HPT results in elevated PTH levels, along with the deterioration of graft function, to maintain normal levels of calcium and phosphate.^5^^,^^6^

Besides, there is a wide variation in the reported prevalence of both persistent and transient HPT after kidney transplantation, which ranges from 10% to 70%.^2^^,^^7^^,^^8^ This variation depends on the diagnostic thresholds used for PTH and calcium levels, as well as the timing to which it refers. Although the most dramatic fall in PTH level occurs in the first 3 months post-KT, HPT is often not diagnosed up to 2 years post-KT, causing increased morbidity from subsequent delays in treatment.^2^^,^^3^

Several studies have linked PHPT to poorer outcomes in KTRs concerning fracture risk,^9^ mortality,^10^ and reduced graft survival.^10^, ^11^, ^12^ Although studies regarding the effect of PHPT on kidney allograft outcome are limited, a stricter association was demonstrated between the presence of PHPT and the reduction of graft function or loss.^13^^,^^14^ The precise pathways triggered by PHPT that are involved in graft dysfunction are poorly understood.

It has been suggested that a potential cause of kidney damage is the deposition of calcium crystals in the tubulointerstitial area because of elevated PTH levels.^13^^,^^14^ This condition, referred to as nephrocalcinosis, can lead to decreased function or graft loss. Although studies on graft biopsies, such as those by Evenepoel et al^13^ and Gwinner et al,^14^ demonstrated a relationship between the deposition of calcium crystals and PTH levels after 3^13^^,^^14^ and 6^14^ months of transplantation, a clear pathophysiological mechanism has not yet been established. Furthermore, some authors have suggested that the decline in graft function may be related to the “vascular hypothesis.” PTH activates adenylyl cyclase, which stimulates the release of renin. PTH has vasodilatory effects on afferent arterioles and constrictive effects on efferent arterioles. The hemodynamic effects of renin mediated by PTH may contribute to the long-term progression of graft failure.^4^^,^^15^^,^^16^

In this study, we aimed to investigate the association between PTH levels at the time of transplantation and the precipitation of calcium crystal deposits (CCDs) found in KTRs protocol biopsies. Second, we sought to assess the role of the excreted fraction of phosphate (FePi) as a mediator in this pathologic process.

## Methods

### Study Population

This is a single-center retrospective study of KTRs who underwent kidney transplantation between July 2021 and August 2022 in the Nephrology Department of IRCCS Policlinico di Sant'Orsola Hospital-University of Bologna. All consecutive KTRs during this period were included, aligning with the implementation of a standardized protocol biopsy program at our center. Patients enrolled had signed informed consent forms to participate in the protocol biopsy program at the time of transplantation; additionally, patients signed a specific consent form for each biopsy performed. All patients who provided informed consent for kidney biopsy were included, with no predefined exclusion criteria. In our transplant center, 3- and 12-month protocol biopsies are a routine procedure and were performed as part of standard clinical practice, independently of the objectives of the present study, which has an entirely noninterventional nature. Among 45 eligible KTRs, 2 were excluded because of graft loss within 3 months and 2 for not providing consent at the time of biopsy.

At the time of transplantation, induction therapy was performed with intravenous methylprednisolone on the day of transplantation (500 mg), followed by 250 and 125 mg on the first and second days after. Subsequently, a daily oral dose of 80 mg was administered and gradually tapered over the first month, in accordance with standard clinical practice. Standard triple immunosuppressive therapy was subsequently started with prednisone 5 mg per day (in 75% of cases), a calcineurin inhibitor, and an antimetabolite. At the time of transplantation, CKD-mineral and bone disorder-related medications were managed according to standard clinical practice, with most treatments, including phosphate binders and calcimimetics, typically discontinued, whereas vitamin D supplementation was continued or adjusted as clinically indicated. Patients with a nonfunctioning graft at 3 months were excluded. Graft biopsies were performed at 3 (T3) and 12 months (T12) after transplantation. The study adheres to the principles of the Declaration of Helsinki and was approved by the ethics committee of the IRCCS Policlinico di Sant’Orsola Hospital-University of Bologna (586/2023/Oss/AOUBo).

### Biochemical Analyses

Laboratory tests were performed at baseline (at the time of transplantation), at discharge (T0), and at the time of graft protocol biopsies (T3 and T12). Discharge was carried out when kidney function was judged stable without any other complications. Biochemical analyses included serum concentrations of creatinine, calcium, phosphate, 25OH-hydroxy-vitamin D, PTH, and alkaline phosphatase, and 24-hour urine concentrations of creatinine, calcium, and phosphate. Serum PTH concentrations were determined using chemiluminescence immunoassay (Beckman Coulter) for intact PTH of the second generation (reference value 15-85 pg/mL). A PTH level above the normal range (PTH levels > 85 pg/mL) after 1 year was defined as PHPT. Estimated glomerular filtration rate (eGFR) was calculated according to the CKD-EPI 2021 formula.^17^ FePi level was calculated using the formula [(urinary phosphate level) × (serum creatinine level)]/[(urinary creatinine level) × (serum phosphate level)]; the same formula was used to calculate fractional excretion of calcium level: [(urinary calcium level) × (serum creatinine level)]/[(urinary creatinine level) × (serum calcium level)].

### Histological Analyses

Percutaneous and ultrasound-guided graft biopsies were performed using a biopsy gun with a 16-gauge needle. Staining was performed on histological preparations on 3 μm thick paraffin sections with hematoxylin-eosin, Periodic acid-Schiff, and silver methenamine methods. The protocol biopsy program did not include Von Kossa staining. Two pathologists assessed the presence of CCDs (oxalate and phosphate levels) in the hematoxylin-eosin slides observed under an optical microscope and with polarized light.

### Statistics

Categorical variables were presented as frequency and percentage; normally distributed continuous variables were expressed as mean ± standard deviation (SD), whereas skewed variables were reported as median and interquartile range [IQR]. All data at baseline were complete. Patients were stratified into 3 groups based on their PTH levels at the time of transplantation^18^: < 150, 150-299, and ≥ 300 pg/mL. Baseline characteristics were compared among groups using the χ^2^ test for categorical variables, analysis of variance for normally distributed continuous variables, and the Kruskal-Wallis test for skewed continuous variables. The association between baseline PTH levels and the probability of having CCDs at any biopsy was assessed using logistic regression models, sequentially adjusted for potential confounders. Confounders were selected a priori based on clinical knowledge and included age, sex, smoking habit, history of diabetes mellitus, dialysis modality and vintage, eGFR values, serum levels of calcium, phosphate, and vitamin D, and therapy with active vitamin D. Therapy with calcimimetics and the type of donor were also identified as possible confounders, but adjustment was not possible because of a violation of the positivity assumption because all patients taking calcimimetics at baseline had CCDs, whereas no CCDs were found in any living donation. The linearity assumption for logistic regression was tested by visual inspection of the scatter plot between PTH levels and the log odds of having CCDs. Moreover, nonlinearity was tested using natural splines. Mediation analysis was conducted using a regression-based approach. In particular, the association between the continuous exposure (PTH) and the continuous mediator (FePi) was modeled using linear regression, and the associations between these and the binary outcome (presence of CCDs) were modeled using logistic regression to compute exact conditional natural direct and indirect effects. All regressions adjusted for sex and age. The mediation analysis was performed using the R package exactmed, in which nested counterfactual probabilities underlying the definition of natural effects are calculated using numerical integration. Confidence intervals were computed by repeating the analysis in 1,000 bootstrapped samples. Given the retrospective and exploratory design of the study, no formal sample size calculation was performed; the sample size was determined by the number of eligible patients during the study period. All analyses were performed using R version 4.1.1.

## Results

### Baseline Characteristics

Forty-five patients were eligible for the study. Of these, 2 were excluded because of graft loss within 3 months posttransplant, and another 2 refused to participate. The mean age of the overall study population (n=41) was 49.6 ± 12.0 years, with 70.7% being men. Baseline PTH levels were < 150, 150-299, and > 300 pg/mL, respectively, in 11, 17, and 13 patients. However, there were no significant differences across patients with different PTH levels. Table 1 summarizes the characteristics of KTRs divided according to baseline PTH levels; patients were categorized based on the cut-off of PTH levels suggested by Kidney Disease Outcomes Quality Initiative guidelines.

**Table 1:** Baseline Characteristics of Patients

General characteristics	All patients (N=41)	Baseline PTH level (pg/mL)			*P*
		< 150 (N=11)	150-299 (N=17)	≥ 300 (N=13)	
Age, y (mean ± SD)	49.6 ± 12	46.3 ± 14	52.9 ± 9.4	48 ± 13	0.32
Sex, males, n (%)	29 (70.7)	7 (63.6)	12 (70.6)	10 (76.9)	0.78
BMI, kg/m^2^ (mean ± SD)	24.3 ± 3.6	23.7 ± 3.7	25 ± 3.7	24.1 ± 3.6	0.64
Ethnicity, n (%)					0.34
Caucasian	33 (80.5)	7 (63.6)	15 (88.2)	11 (84.6)	
Others	8 (19.5)	4 (36.4)	2 (11.8)	2 (15.4)	
Medical history, n (%)					
Diabetes mellitus	3 (7.3)	0 (0.0)	2 (11.8)	1 (7.7)	0.51
Primary kidney disease					0.38
Glomerulonephritis	15 (36.6)	5 (45.5)	6 (35.3)	4 (30.8)	
Diabetic nephropathy	2 (4.9)	0 (0.0)	1 (5.9)	1 (7.7)	
ADPKD	5 (12.2)	1 (9.1)	4 (23.5)	0 (0.0)	
CAKUT	8 (19.5)	1 (9.1)	2 (11.8)	5 (38.5)	
Unknown	11 (26.8)	4 (36.4)	4 (23.5)	3 (23.1)	
PTX (either total or subtotal)	7 (17.1)	4 (36.4)	2 (11.8)	1 (7.7)	0.13
Dialysis, n (%)					
Modality					0.62
None (pre-emptive)	7 (17.1)	1 (9.1)	2 (11.8)	4 (30.8)	
HD	30 (73.2)	9 (81.8)	13 (76.5)	8 (61.5)	
PD	4 (9.8)	1 (9.1)	2 (11.8)	1 (7.7)	
Vintage, mo, median (IQR)	32 (9, 62)	24 (8, 68)	48 (9, 62)	20 (17, 56)	*0.69*
Mineral biomarker levels, median (IQR)					
Total calcium level (mg/dL)	9.4 (9-9.8)	9.4 (8.6-9.8)	9.6 (9-9.9)	9.2 (8.6-9.6)	0.25
Phosphate level (mg/dL)	4.9 (4.2-6)	4.9 (3.8-6.3)	4.9 (4.6-5.8)	5.1 (4.1-6)	0.86
25 (OH)-vitamin D level (ng/mL)	12 (7-15)	12 (7-13.5)	13 (8-16)	9 (7-15)	0.80
PTH level (pg/mL)	241 (142- 333)	106 (58-131)	238 (213-255)	412 (379-463)	**< 0.001**
ALP level (U/L)	70 (61-94)	61 (56.5- 81.5)	70 (65-78)	75 (66-100)	0.34
CKD-MBD medications, n (%)					
Nutritional vitamin D	6 (14.6)	2 (18.2)	1 (5.9)	3 (23.1)	0.39
VDRA	28 (68.3)	8 (72.7)	11 (64.7)	9 (69.2)	0.90
Calcimimetics	7 (17.1)	1 (9.1)	2 (11.8)	4 (30.8)	0.28
Cinacalcet	4 (9.8)	1 (9.1)	0 (0.0)	3 (23.1)	0.11
Etelcalcetide	3 (7.3)	0 (0.0)	2 (11.8)	1 (7.7)	0.51
Phosphate binders^a^	34 (82.9)	10 (90.9)	14 (82.4)	10 (76.9)	0.66
Calcium-based	19 (46.3)	6 (54.5)	5 (29.4)	8 (61.5)	0.18
Calcium-free	28 (68.3)	8 (72.7)	13 (76.5)	7 (53.8)	0.39
Transplant characteristics, n (%)					
Donor, deceased	27 (65.9)	8 (72.7)	10 (58.8)	9 (69.2)	0.72
Single kidney	36 (87.8)	10 (90.9)	14 (82.4)	12 (92.3)	0.67
DCD	4 (9.8)	1 (9.1)	1 (5.9)	2 (15.4)	0.68

There were no missing data at the baseline.

Bold indicates statistically significant value (*p* < 0.05).

Abbreviations: ADPKD, autosomal dominant polycystic kidney disease; ALP, alkaline phosphatase; CAKUT, congenital anomalies of the kidney and urinary tract; CKD, chronic kidney disease; DCD, donation after circulatory death; HD, hemodialysis; MBD, mineral and bone disorder; PTX, parathyroidectomy; PD, peritoneal dialysis; PTH, parathyroid hormone; VDRA, Vitamin D receptor activator.

a Some patients received both calcium-based and calcium-free phosphate binders. Some patients received both calcium-based and calcium-free phosphate binders; therefore, the sum of the two subcategories (n = 19 and n = 28) exceeds the total number of patients receiving phosphate binder therapy (n = 34).

### Biochemical Findings

At baseline, the median PTH level was 241 (IQR, 142-333) pg/mL. After transplantation, there was a significant reduction in PTH, although a complete normalization was not always achieved (Figure S1). Indeed, 48% of patients still had PTH levels above the upper limit of normality (85 pg/mL) after 1 year from transplant. As expected, a significant transient decrease in calcium levels and a persistent decrease in phosphate levels were also observed after transplantation (Figure S1).

### Histological Findings

**Figure 1:**
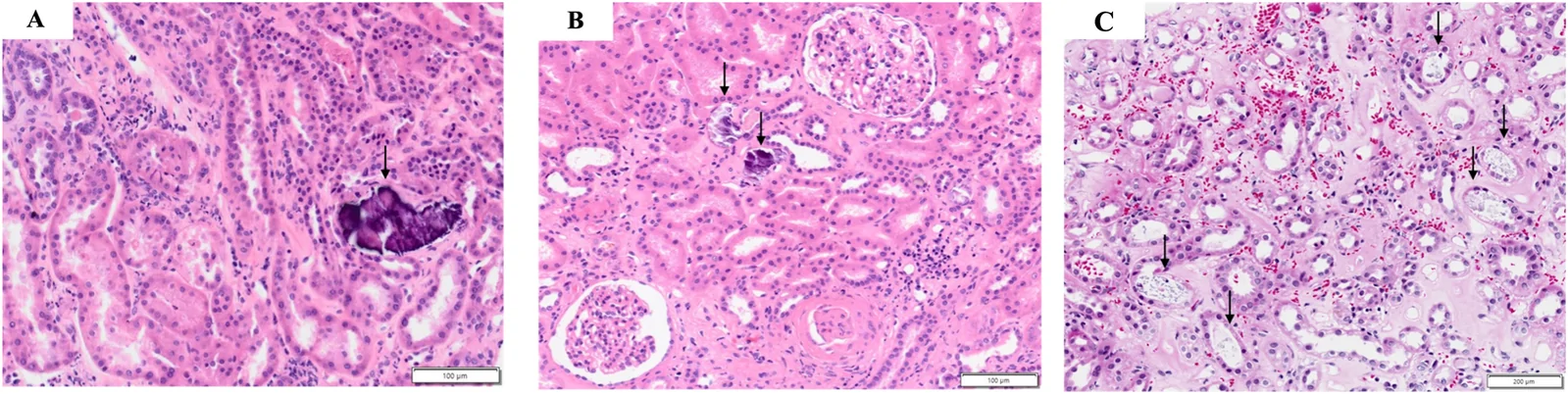
Crystals of calcium phosphate at T3 (A) and T12 (B). Crystal of calcium oxalate at T12 (C). Hematoxylin-eosin (original magnification 400×). Abbreviations: T3, graft biopsies were performed at 3 months after transplantation; T12, graft biopsies were performed at 12 months after transplantation.

Table 2 summarizes the main histological findings at T3 and T12. At T3, 39 of 41 patients underwent biopsy because 2 declined to renew informed consent; at T12, 29 biopsies were performed, with 1 patient deceased and 11 refusing the procedure. At T3, CCDs were observed in 13 samples out of 39 (33%). At T12, CCDs were identified in 13 samples out of 29 (45%): in 7 cases, the CCDs were observed for the first time, whereas in 6, they were already present in a previous biopsy. At both time points, CCDs were predominantly composed of calcium phosphate and localized in the tubule. At T3, most patients exhibited only 1 single CCD, with only 4 cases of multiple CCDs, whereas at T12, 7 biopsies showed a single CCD and 6 showed multiple CCDs. Figure 1 presents the images of CCDs at T3 and T12.

**Table 2:** Histological Findings

Parameters	Biopsy T3 (N=39)	Biopsy T12 (N=29)
Time from transplant (d), median (IQR)	105 (92-142)	451 ( 404-463)
Sample dimension (mm^2^), median (IQR)	4.35 (2.95-5.39)	4.03 (3.00-5.52)
eGFR (mL/min/1.73 m^2^), mean (±SD)	51.8 ± 20.6	54.4 ± 20.4
Deposits, n (%)		
Presence	13 (33)	13 (45)
Novel finding	-	7 (54^b^)
Already known	-	6 (46^b^)
Type		
Calcium phosphate	12 (92^a^)	11 (85^a^)
Calcium oxalate	1 (8^a^)	2 (15^a^)
Number		
1	9 (70^a^)	7 (54^a^)
2	2 (15^a^)	5 (38^a^)
3+	2 (15^a^)	1 (8^a^)
Location, n (%)		
Tubular	11 (85)	6 (46^a^)
Interstitial	2 (15)	7 (54a)

Abbreviations: eGFR, estimated glomerular filtration rate.

a % of those with deposits.

b in those with deposits.

### Association Between Baseline PTH Levels and the Probability of Having CCDs

**Figure 2:**
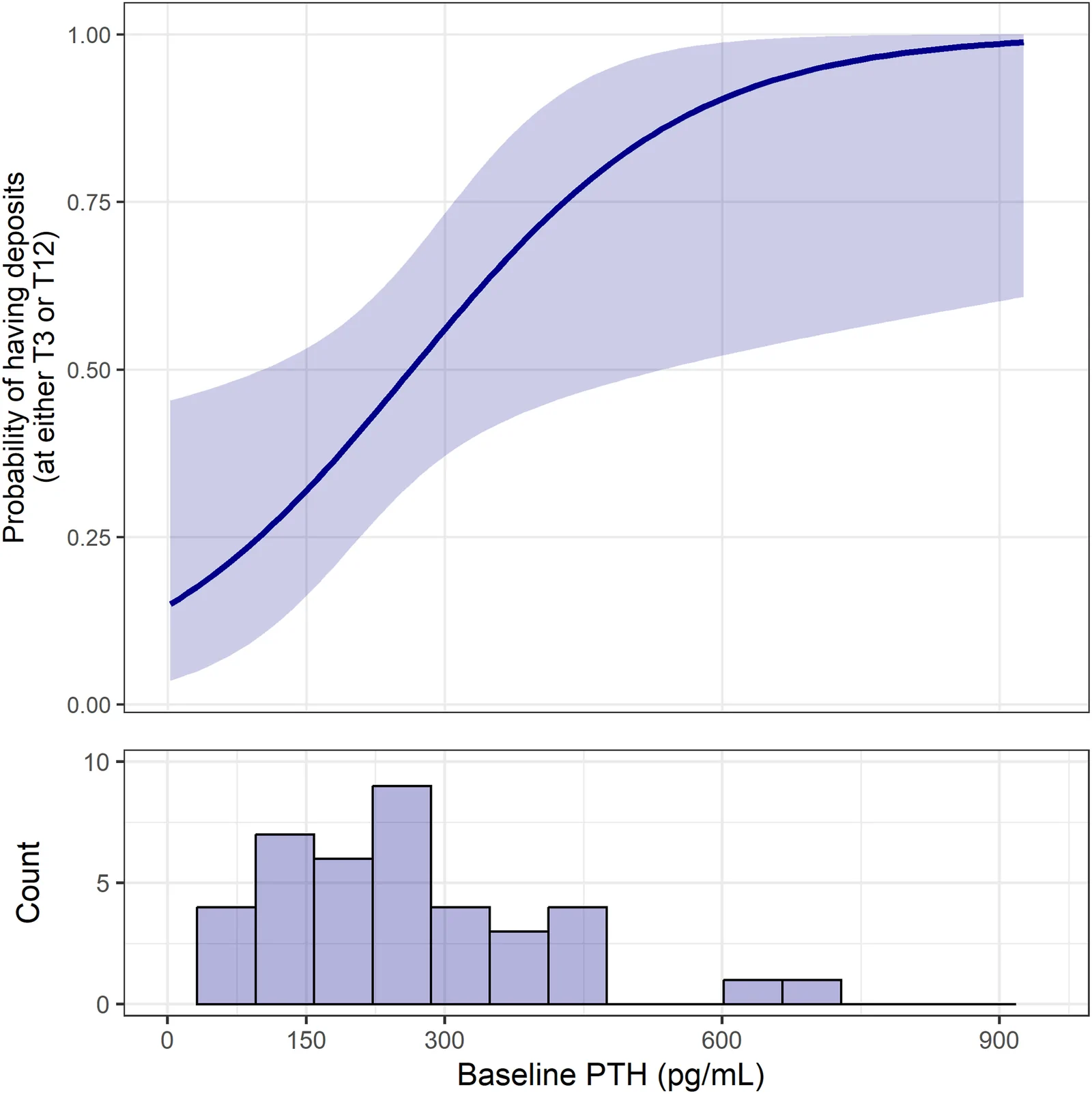
Association between baseline parathyroid hormone (PTH) levels and the probability of having calcium crystal deposits (CCDs) at any biopsy. Upper panel: association between baseline PTH levels and the predicted probability of CCDs in graft biopsies, as estimated using logistic regression, with 95% confidence interval (CI) shown as the shaded area. Lower panel: histogram illustrating the distribution of baseline PTH levels in the study cohort, showing a wide interindividual variability.

As shown in Figure 2, a significant linear association was found between baseline PTH levels and the probability of having CCDs in protocol biopsies. In the fully adjusted model, a 10 pg/mL increase in PTH values was associated with a 20% higher risk of CCDs (odds ratio, 1.20; confidence interval, 1.08-1.45; *P* = 0.01).

### Biochemical Profile in Patients With and Without CCDs

Table S1 presents kidney function and mineral metabolism parameters at T3 and T12, stratified by the presence of CCDs. Although no significant differences were observed for eGFR values, serum creatinine levels of calcium, phosphate, 25(OH)-vitamin D, or alkaline phosphatase, patients with CCDs exhibited significantly higher FePi and fractional excretion calcium values, as well as elevated PTH concentrations, compared with those without CCDs. These findings suggest a greater disruption of phosphate-calcium homeostasis in the subgroup with complications.

### The Role of FePi in the Deposition of CCDs

**Figure 3:**
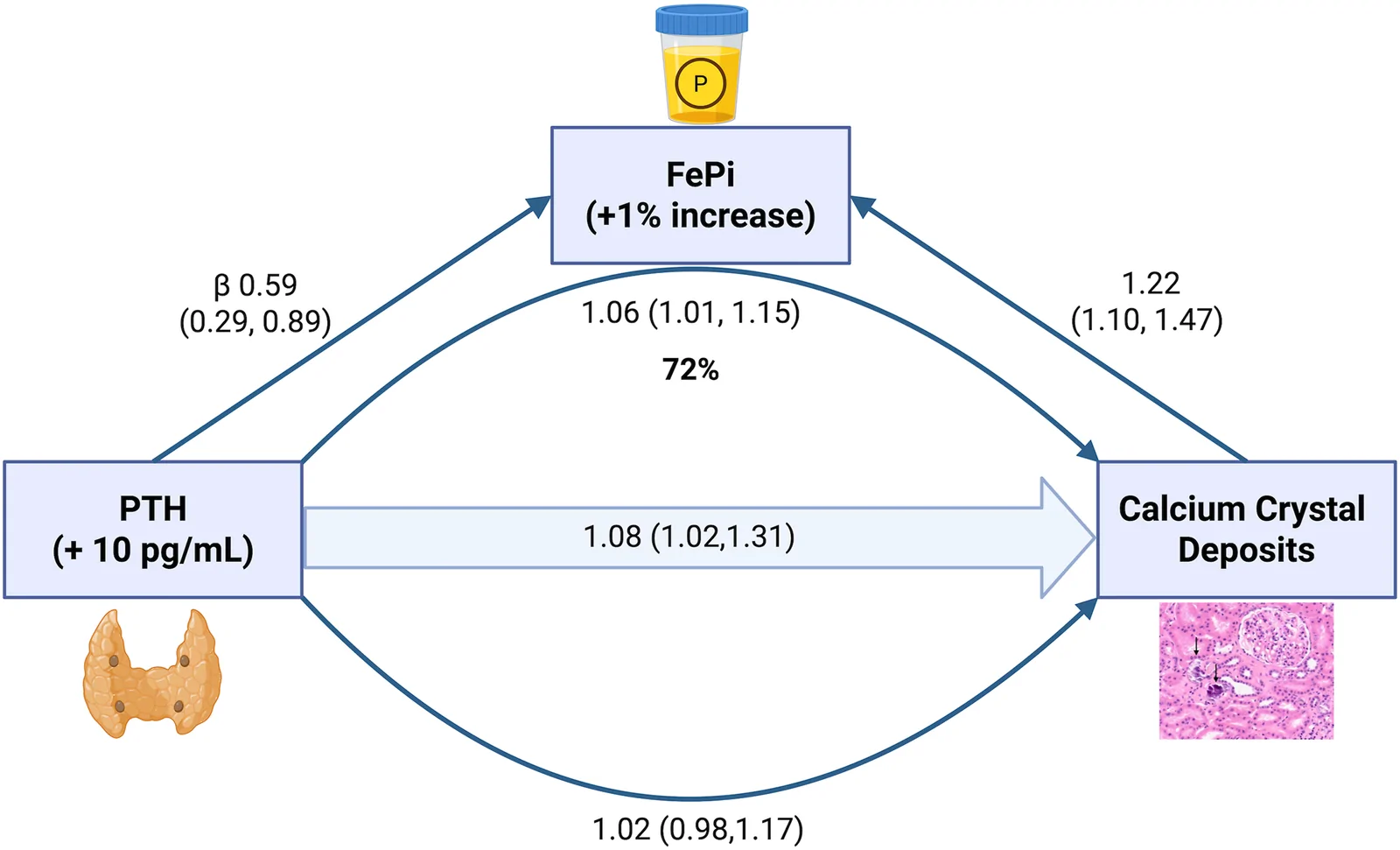
Mediation analysis of the association between parathyroid hormone (PTH) levels at transplantation and calcium crystal deposits (CCDs), with fractional excretion of phosphate (FePi) as mediator. Each 10 pg/mL increase in PTH level was associated with an 8% higher risk of CCDs (hazard ratio [HR], 1.08; 95% CI, 1.02-1.31). Of this effect, 72% was mediated by FePi level (HR, 1.06; 95% CI, 1.01-1.15), whereas the direct effect of PTH level independent of FePi level was modest (HR, 1.02; 95% CI, 0.98-1.17).

The FePi level was evaluated as a potential mediator in the deposition of calcium crystals. The total effect of PTH on the probability of CCDs corresponded to a hazard ratio (HR) of 1.08, indicating an 8% increase in risk for each 10 pg/mL increase in PTH levels at the time of transplantation. Higher PTH levels at transplantation were also associated with higher FePi values at discharge, with each 10 pg/mL increase in PTH level corresponding to a mean increase of +0.59% in FePi value. In turn, each 1% increase in FePi values at discharge was independently associated with a 22% higher probability of CCDs. Mediation analysis revealed that approximately 72% of the total effect of PTH on CCD risk was mediated through FePi (hazard ratio, 1.06), whereas the direct effect of PTH level, independent of FePi value, was smaller (hazard ratio, 1.02; Figure 3).

### Long-term Graft Function

At 24 months post-transplantation, eGFR values were comparable between patients with and without CCDs, consistent with findings at 12 months and with largely overlapping distributions between the 2 groups.

## Discussion

In this retrospective observational study, we showed that elevated PTH levels at the time of kidney transplantation are associated with a higher risk of developing CCDs in the graft, and that a major part of this effect is mediated by increased FePi levels. Specifically, for every 10 pg/mL increase in baseline PTH level, the risk of CCDs increased by 20%, and approximately 72% of this effect was mediated by FePi. These findings suggest a key role for phosphate handling in the pathophysiology of mineral deposition in the allograft.

The results highlighted a high prevalence of CCDs in graft protocol biopsies, with rates of 33% at 3 months and 45% at 12 months posttransplantation. In a comparable study, Evenepoel et al^13^ reported a similar prevalence of 31.8% at 3 months in a cohort of 201 KTRs.^13^ Conversely, Gwinner et al^14^ observed lower prevalences of CCDs—6.1% at 6 weeks and 17.8% at 6 months—in 586 protocol graft biopsies.^14^ These differences may be related to the different timing of biopsy procedures and differences in the criteria for identifying CCDs, such as the inclusion of calcium oxalate crystals in our study. Regarding the relationship between mineral disturbances and CCDs, we found that pretransplant PTH levels significantly increased the odds of developing CCDs and that also at the time of the biopsy patients with CCDs had significantly higher PTH and FePi levels compared with those without CCDs. Similarly, Evenepoel et al^13^ observed that nephrocalcinosis was associated with high levels of PTH, FePi, and serum calcium, as well as low phosphate levels. Gwinner et al^14^ found that patients with CCDs had high PTH levels at the time of the biopsy, independent of serum creatinine and calcium levels. In both studies, the persistence of high PTH values in the early posttransplant period was associated with hypophosphatemia and hypercalcemia, which were caused, respectively, by kidney phosphate wasting and calcium release from the skeleton. These findings suggest that deranged mineral metabolism is involved in the pathogenesis of nephrocalcinosis.^7^ In this context, it is important to note that the increased risk of CCDs observed in our study does not appear to be driven by excessive phosphate intake but rather by maladaptive phosphate wasting secondary to elevated PTH levels. This has relevant clinical implications because it suggests that a more aggressive use of phosphate binders in the early posttransplant period may not be appropriate. Instead, our findings highlighted the importance of optimizing HPT management before transplantation to prevent persistent disturbances in phosphate level handling. Similarly, strict dietary phosphate restriction alone is unlikely to address the underlying pathophysiological mechanism leading to crystal deposition in this population. Furthermore, these results provided novel insights into both the temporal and spatial progression and the underlying pathophysiological mechanisms of CCDs. After 3 months, most CCDs were located within the tubules, whereas after 12 months, their distribution had shifted, with 46% still in the tubule and 54% now located in the interstitium. This pattern confirms the temporal sequence proposed by Evenepoel et al,^13^ who hypothesized that CCDs initially deposit in the tubules, where they are retained and internalized, and subsequently translocate to the interstitium.^13^ In addition, the mediation analysis revealed that approximately 72% of the effect of baseline PTH levels on CCDs is mediated by increased FePi levels, underscoring the central role of phosphate wasting in this pathologic sequence. In our cohort, CCDs were predominantly composed of calcium phosphate, whereas calcium oxalate deposits were only rarely observed. Given their low prevalence, it is unlikely that calcium oxalate crystals significantly influenced the overall findings. Moreover, these 2 types of deposits reflect different pathophysiological mechanisms, with calcium phosphate deposition being more closely associated with disturbances in PTH levels and phosphate levels handling, which were the primary focus of our study. This distinction further supports the relevance of the PTH-phosphate axis in the development of CCDs observed in our study.

In our study, the prevalence of PHPT at 1 year was 48%. This aligns with the findings of Crepeau et al,^12^ who reported PHPT (defined as PTH levels > 70 pg/mL) in 60.9% of 824 KTRs at 1 year, and Isakov et al,^4^ who found that 37.7% of KTRs had PTH levels > 150 pg/mL at 3 months after transplantation.^4^

Several studies have shown that high PTH levels increase the risk of graft loss and mortality.^4^^,^^10^, ^11^, ^12^^,^^19^ However, the mechanisms by which PTH levels may negatively affect graft function are still debated. Proposed explanations include tubulointerstitial deposition of CCDs,^13^^,^^14^ the promotion of interstitial fibrosis,^20^^,^^21^ and hemodynamic alterations because of the effect of PTH levels on renal arterioles.^4^^,^^22^ Regarding the link between the presence of CCDs in graft biopsies and the worsening of kidney function, Gwinner et al^14^ observed poorer graft outcomes after 1 year in patients with CCDs and elevated PTH levels,^14^ whereas the same association was not described by Evenepoel et al.^13^ Similarly, in our study, we did not observe significant differences in kidney function after 12 months from transplantation in patients with and without CCDs. This finding was consistent at 24 months posttransplantation, with no clear difference in graft function between the 2 groups. However, this may be because of the limited sample size and short follow-up duration. The role of posttransplant HPT in promoting fibrosis was demonstrated in 2 retrospective studies, which showed that PTH levels are independently associated with the severity of interstitial fibrosis observed in graft biopsies.^20^^,^^21^ In 1 of these studies, Schwarz et al^21^ also found a correlation between fibrosis and the presence of nephrocalcinosis in graft biopsies, suggesting that this could be a risk factor for the development of chronic allograft nephropathy, defined by the Banff Conference as “interstitial fibrosis and tubular atrophy without evidence of specific aetiology.”^23^ Experimental evidence further supports a role for PTH in the progression of interstitial fibrosis. For instance, PTH has been shown to induce epithelial-to-mesenchymal transition in human renal proximal tubular cells via the Wnt/β-catenin signalling pathway.^24^ Finally, it has been suggested that PTH plays a role in regulating kidney filtration. The so-called “vascular hypothesis” proposes that PTH modulates glomerular hemodynamics by acting on vascular receptors in renal arterioles. PTH significantly increases renal plasma flow by approximately 50% and slightly raises the glomerular filtration fraction by around 10%, suggesting a more pronounced effect on the efferent arterioles.^22^ In animal studies, PTH has been shown to modify filtration by vasodilating afferent arterioles and constricting efferent arterioles, which reduces the ultrafiltration coefficient. Furthermore, PTH stimulates renin release, which may offer short-term protection but can lead to long-term graft damage because of hyperfiltration. Additionally, PTH may induce structural changes in blood vessels, making arteries less responsive to vasodilation, as a result of its action on vascular cells such as smooth muscle, endothelial cells, and cardiomyocytes.^4^^,^^22^^,^^25^

Previous studies have also reported that PTH and fibroblast growth factor 23 (FGF23) act synergistically to promote hypercalcemia, hypercalciuria, and elevated phosphate wasting during the first year posttransplantation.^3^^,^^7^^,^^26^ Moreover, recent studies in mice have suggested that excessive phosphate intake, exceeding the number of functioning nephrons, elevates FGF23 levels, reducing phosphate reabsorption and increasing phosphate concentration in the tubular lumen. At specific phosphate levels, microscopic calcium phosphate particles were observed, which induced damage to the proximal tubule cells by binding to the Toll-like receptor 4 expressed on them.^27^ The persistence of the damage led to increased interstitial fibrosis, further reducing the number of functioning nephrons and advancing CKD.^27^ Adapting the study to humans, it was hypothesised that FGF23 levels begin to rise when phosphate concentrations in the proximal tubular fluid exceed 2.32 mg/dL and that tubulointerstitial damage develops at FGF23 levels of approximately 53 pg/mL.^27^ Wolf et al,^7^ in a multicenter, prospective, observational cohort study of 246 KTRs, observed that FGF23 levels decrease rapidly in the first 3 months after transplantation, from 492.1 ± 25.7 to 34.1 ± 10.7 pg/mL in KTRs with PTH levels above 300 pg/mL at the moment of the transplant and from 355.6 ± 29.3 to 16.9 ± 1.3 pg/mL in KTRs with PTH levels from 65 to 300 pg/mL. High FGF23 levels were correlated with higher PTH and lower phosphate levels in the first 3 months posttransplant.^7^ Although FGF23 level was not available in our dataset, the strong association between PTH and FePi levels observed in our mediation analysis supports a central role for disturbed phosphate metabolism in the development of CCDs. It is plausible to speculate that if the pretransplant PTH level is not adequately controlled, persistent alterations in mineral metabolism in KTRs may contribute to early nephrocalcinosis and subsequent graft loss, reflecting what happens in the earliest phase of CKD.

This study has some limitations, including its retrospective design, small sample size, relatively short observation period, and the absence of FGF23 measurements. Nonetheless, it offers new evidence indicating that elevated PTH levels before transplantation—and not only during the early posttransplant period—may contribute to nephrocalcinosis through mechanisms involving phosphate wasting. Another limitation is the lack of Yasue or Von Kossa staining, which could provide more sensitive detection of calcium deposits. In our study, crystal identification was based on hematoxylin-eosin staining combined with polarized light microscopy and expert pathologic evaluation, a method commonly adopted in similar studies. Although the use of more sensitive staining techniques might have increased the detection rate of calcium crystals, any potential misclassification is likely to be nondifferential and therefore would be expected to attenuate, rather than inflate, the observed associations. Another limitation is that the potential impact of posttransplant medications, such as calcineurin inhibitors on phosphate handling and mineralocorticoid receptor antagonists on fibrosis, was not specifically considered. Because of the limited sample size, including these variables in the analytical models was not feasible, and residual confounding related to these factors cannot be ruled out.

In conclusion, we observed a high prevalence of CCDs in KTRs with altered mineral metabolism, particularly those with elevated PTH and FePi levels. Importantly, our data suggest that elevated pretransplant PTH levels are an independent risk factor for developing nephrocalcinosis and that this effect is largely mediated by increased FePi levels. These findings support the need for careful pretransplant evaluation and management of secondary HPT to prevent posttransplant complications.
