# Supplementary material for: Calcium Crystal Deposits in Kidney Allografts: The Interplay of PTH Levels and Phosphaturia
Source: Kidney Med. 2026 Jul 11;8(9):101464. doi: 10.1016/j.xkme.2026.101464 (PMC13524679; doi:10.1016/j.xkme.2026.101464)
Supplement: Supplementary file 1 [file mmc1.pdf]

Figure S1. Laboratory parameters at baseline, T0, T3 and T12 (overall). *Abbreviations: eGFR, estimated Glomerular Filtration Rate; PTH, Parathyroid Hormone; ALP, Alkaline phosphatase; Fe_{Ca}, Fractional excretion of calcium; Fe_{Pi}, Fractional excretion of phosphate*

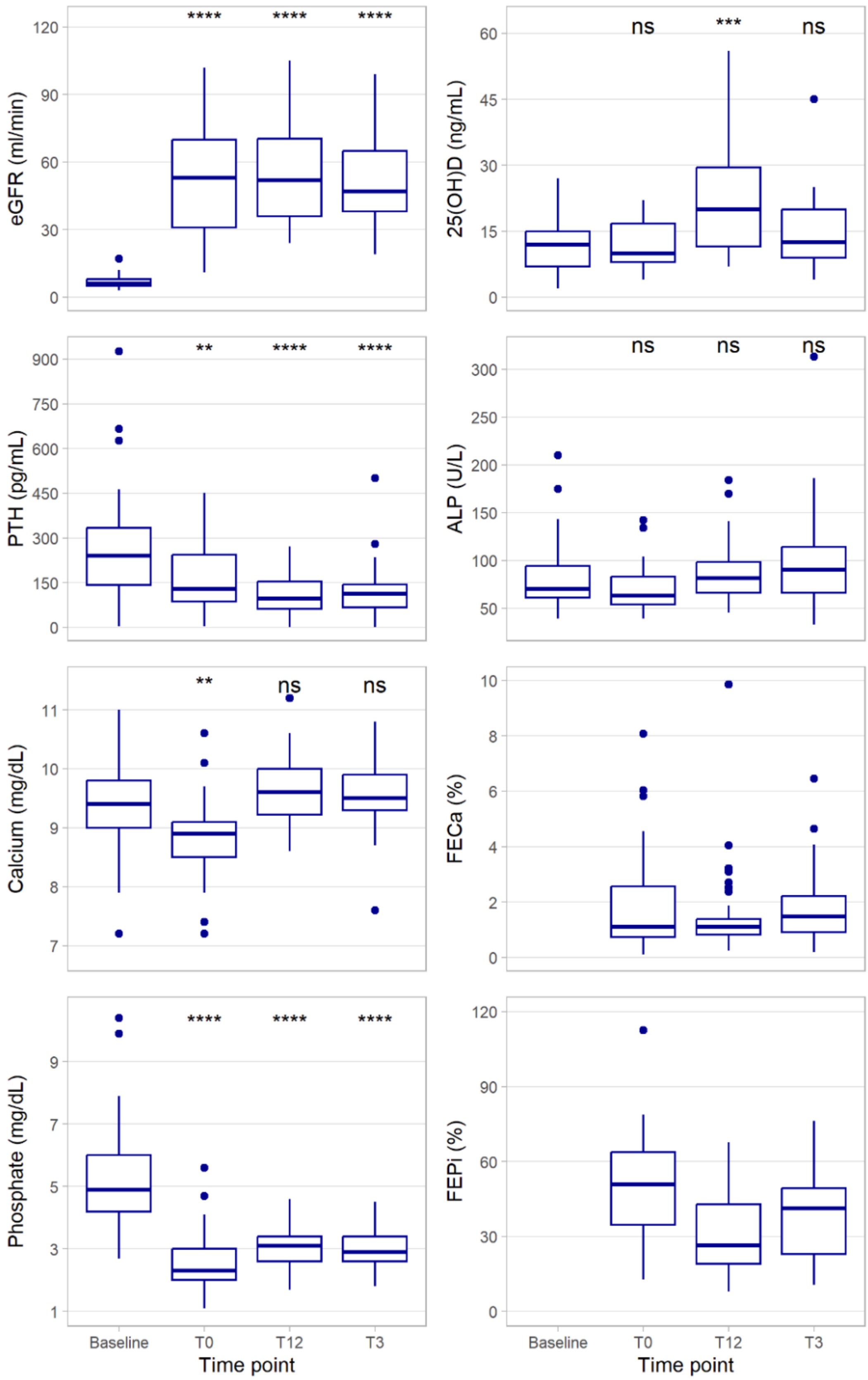

Table S1. Renal function and mineral metabolism parameters at T3 and T12 in patients with and without CCDs.

	T3				T12			
	**Overall** N = 39	**Without CCDs** N = 26	**With CCDs** N = 13	**p**	**Overall** N = 29	**Without CCDs** N = 16	**With CCDs** N = 13	**p**
Creatinine (mg/dL)	1.6 [1.2, 2.0]	1.6 [1.3, 2.1]	1.8 [1.1, 2.0]	0.64	1.5 [1.3, 1.9]	1.7 [1.4, 1.9]	1.5 [1.1, 1.8]	0.43
eGFR (ml/min/1.73 m^2)	50.4 ± 19.9	49.9 ± 19.5	51.5 ± 21.5	0.82	52.6 ± 19.5	52.7 ± 19.5	52.5 ± 20.1	0.98
Total calcium (mg/dL)	9.6 ± 0.6	9.5 ± 0.6	9.7 ± 0.6	0.39	9.6 ± 0.6	9.6 ± 0.5	9.7 ± 0.8	0.82
Serum Phosphate (mg/dL)	2.9 ± 0.7	3.0 ± 0.7	2.8 ± 0.6	0.47	3.1 ± 0.6	3.1 ± 0.7	3.0 ± 0.5	0.66
Urine Phosphate (mg/day)	598.5 ± 235.5	510.0 ± 223.5	776.0 ± 142.5	*<0.001*	455.9 ± 245.8	268.5 ± 121.5	625.5 ± 202.5	*<0.001*
FePi (%)	39.9 ± 15.7	34.0 ± 14.9	51.7 ± 9.5	*<0.001*	32.4 ± 16.5	23.0 ± 13.2	43.9 ± 12.5	*<0.001*
FeCa (%)	1.6 [0.9, 2.3]	1.6 [0.9, 2.3]	1.6 [1.1, 2.1]	0.93	1.1 [0.9, 1.3]	1.2 [1.0, 1.7]	1.0 [0.7, 1.2]	0.05
25(OH)-vitamin D (ng/mL)	12 [9, 18]	10 [9, 16]	16 [10, 20]	0.19	20 [12, 32]	25 [16, 32]	15 [10, 23]	0.18
PTH (pg/mL)	123 [75, 144]	112 [54, 139]	134 [113, 165]	0.04	100 [64, 163]	69 [53, 113]	125 [100, 171]	0.04
ALP (U/L)	92 [68, 115]	91 [62, 102]	114 [71, 148]	0.12	76 [61, 91]	76 [64, 93]	75 [56, 88]	0.43
